# Micronuclei formation in liver fibrosis samples from patients infected by hepatitis C virus

**DOI:** 10.1590/S1415-47572010005000061

**Published:** 2010-09-01

**Authors:** Terezinha M. B. de Almeida, Regina Maria C. Leitão, Flair J. Carrilho, Shigueko Sonohara

**Affiliations:** 1 Disciplina de Oncologia, Departamento de Radiologia e Oncologia, Faculdade de Medicina, Universidade de São Paulo, São Paulo, SPBrazil; 2 Laboratório de Anatomia Patológica Diagnóstika, São Paulo, SPBrazil; 3 Disciplina de Gastroenterologia Clínica-Hepatologia, Departamento de Gastroenterologia, Faculdade de Medicina, Universidade de São Paulo, São Paulo, SPBrazil

**Keywords:** micronuclei, hepatocytes, chronic hepatitis C, fibrosis progression, HCV-patients

## Abstract

Genetic research on fibrosis outset and its progression in chronic hepatitis (CH) by hepatitis C virus (HCV) are limited. The lack of cytogenetic data led us to investigate the presence of micronuclei (MNi), as a sign of genomic damage. Hepatocytes of hepatic parenchyma from 62 cases diagnosed with CH associated with HCV and displaying different degrees of fibrosis (F1-F4) were analyzed. These data were compared to 15 cases without fibrosis (F0). Twelve healthy liver parenchyma samples were included as control. All samples were obtained from paraffin-embedded archival material. Micronucleated hepatocytes (MN-Heps) were analyzed through Feulgen/Fast-green staining. Results showed that the rates of MN-Heps in the F4 group were statistically significant (p < 0.05) and higher than those in the control group. Like results were also obtained on comparing F4 with F0, F1, F2 and F3 cases. Conversely, differences were not significant (p > 0.05) on comparing F0, F1, F2, F3, one against the other, as well as individual versus control. Although chromosomal losses in CH were detected, it was shown that liver parenchyma with fibrosis in the initial stages (F1-F3) cannot be considered cytogenetically abnormal.

## Introduction

Chronic infection by hepatitis C virus (HCV) is both slow and insidious. 80% of HCV individuals are prone to infection worldwide. From these, over 30% develop chronic liver diseases, including cirrhosis, a major risk for developing hepatocellular carcinoma (HCC) (Thomson and Finch, 2005). This possibility is about 17 times higher in HCV infected patients than in uninfected (Donato *et al.* 2001). However, the evolution of chronic hepatitis (CH) varies in HCV infected patients, due, not only to the time of infection, but also to the extent of fibrosis generated| by hepatic parenchyma during the necro-inflammatory process (Kiyozawa, 2002; But *et al.*, 2008). The elimination of hepatocytes in CH/HCV during the necro-inflammatory process is followed by accelerated cell division, thereby leading to various grades of fibrosis (F1-F4). In spite of this type of cell proliferation induced by chronic lesion, the liver may still be considered functionally competent (Callea *et al.*, 1991). The intense hepatocyte turnover may make all cells susceptible to genetic variation, this including cytogenetic instability, thus creating propitious conditions for the development of the various stages of hepatocarcinogenesis (Kitamura *et al.*, 1998).

There are no data available on chromosomal instability from the onset of the fibrosis progression caused by virus infection in human beings. However, cytogenetic changes in micronucleated hepatocytes (MN-Heps) through chromatin loss were found in CH/HCV-F4 in regenerative cirrhotic nodules without atypia (Almeida *et al.*, 2004; Guido *et al.*, 2008; Kim *et al.*, 2009). Micronuclei may originate either from acentric fragments, resulting from chromosomal/chromatid breaks, or from whole chromosomes, unable to migrate with the remainder chromosomes in anaphase, and being included in one or more secondary nuclei in daughter cells. Thus, MN assays can be used to evaluate clastogenic and aneugenic effects (Heddle *et al.*, 1991; Fenech, 2000). The hypothesis was that micronuclei, as the result of chromosome breakage/anaphase lagging, might occur in CH/HCV at the start of the fibrotic process, and increasing in cirrhosis and in HCC. To check this hypothesis, MN-Hep frequencies inside CH/HCV liver parenchyma with (F1-F4) or without fibrosis (F0) and with normal parenchyma (NP), were examined.

## Material and Methods

Liver tissue samples from 89 individuals, both male and female (age 18-60), were analyzed. Study material consisted of hepatocytes in 77 samples from CH|HCV patients without fibrosis (F0), and parenchyma with fibrosis in the initial (F1, F2, F3) and final (F4) stages, as well as samples from twelve healthy donors - NP (control group). All the samples were retrospective from 2002 to 2007, and collected from the Laboratório de Anatomia Patológica Diagnóstika, São Paulo, Brazil. According to clinical references, the patients had not been previously exposed to any kind of etiological agent, including those known as mutagenic.

###  Tissue samples

CH/HCV samples were separated into the following groups: F0 (15); F1 (14); F2 (16); F3 (15); F4 (17), plus 12 control individuals. Retrospective studies were performed on liver samples fixed in 10% formalin and embedded in paraffin blocks. For light microscopy, each sample in paraffin blocks was cut into 3-5 μm-thick sections and stained with hematoxylin-eosin in order to choose the areas to be studied. The fibrosis-stage for investigation in each case was determined by the histological characteristics imputed by both the METAVIR system (The French METAVIR Cooperative Study Group, 1994) and International Working Party (1995). These are: F0- without fibrosis; F1- mild fibrosis; F2- moderate fibrosis; F3- severe fibrosis and F4- cirrhosis in regenerative nodules (RNs) without atypia. The normal hepatic parenchyma was chosen at random. After establishing these areas the samples were prepared for MN-Heps analysis.

###  Detection of micronuclei

Slides were stained by the Feulgen reaction and counterstained with fast-green, as described by Almeida *et al.* (2004). Each slide was code-labeled, so as to avoid observer bias, and examined at 1250x magnification with a standard optical microscope (Olympus BX-50). The number of hepatocytes scored was usually 2000 per sample. The same criteria described by Almeida *et al.* (2004) were used for evaluating MN-Heps. This cytogenetic evaluation was carried out with 178,000 mononucleated hepatocytes from the following chronic hepatitis parenchyma (CHP) groups: 30,000 (F0); 28,000 (F1); 32,000 (F2); 30,000 (F3) and 34,000 (F4), besides 24,000 with normal parenchyma (NP). The results were all expressed according to the number of MN-Heps per 1000 hepatocytes (‰).

###  Statistical analysis

Since data were not normally distributed, differences among groups were checked through nonparametric analysis of variance (Kruskall-Wallis test), followed by a multiple comparisons test in order to locate those comparisons responsible for significance of the test. Commercial (SPSS Inc., Chicago, IL, USA) and public domain (Online R Development Team, 2009) softwares were used for all mentioned statistical analyses.

## Results

**Figure 1:**
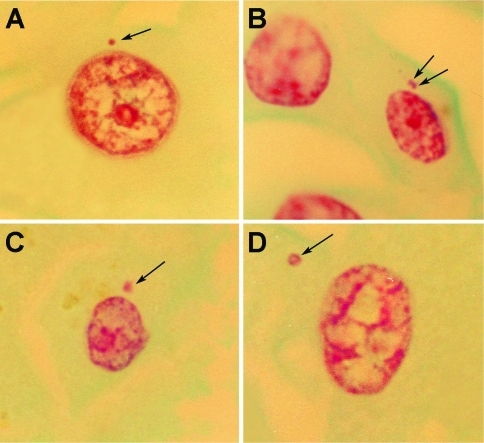
Photomicrographs of micronucleated hepatocytes detected with Feulgen-fast-green staining in interphasic nuclei from chronic hepatitis-HCV parenchyma and normal liver tissues. Irrespective of analyzed area and even in normal parenchyma, most cells presented only one micronucleus (Panels A, C, D). Panel B: a micronucleated hepatocyte with two micronuclei. Variation in size: hepatocytes with (A) small micronucleus, (B) two small micronuclei, (C) medium micronucleus, (D) large micronucleus. Arrows indicate micronuclei.

**Figure 2:**
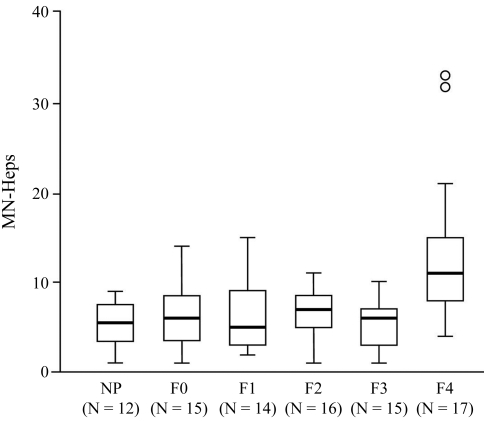
MN-heps data on the progression of fibrosis levels F0 to F4 and normal parenchyma (NP). Boxes indicate medians, whiskers and minimum and maximum values, extending to 1.5 times the interquartile range; circles represent outliers.

The distribution of micronuclei (MNi) per hepatocyte in the F0, F1, F2, F3 and F4 groups, irrespective of parenchymal area, and even in the NP (control group) indicated only one MN in most cells (95.6%), as shown in Figure 1A, B, C. In the F0 group, in about 22% of the cases, incidence was one or two MNi per cell (Figure 1D), the same occurring in the F3 and F4 groups, although, in the control group, in 30% of the cells, this was one to three MNi. In most NP cells, incidence was one MN per cell, although 6 hepatocytes with two MNi each were also found. The MN-Heps evaluated in the parenchyma of each group were of different sizes. In F0-F3 groups size mostly varied from medium (1/10 diameter of the main nucleus) to large (1/5) (Figure 1C, D). In F4, most MNi small-sized (1/15), even when there were two (Figure 1A, B). MN-Hep frequencies were obtained from CHP in different stages of fibrosis (F1, F2, F3, F4), as well as parenchyma without fibrosis (F0) and in NP. Median values (range) were distributed, as follows: F0: 6 (1-14), N = 15; F1: 5 (2-15), N = 14; F2: 7 (1-11), N = 16; F3: 6 (1-10), N = 15; F4: 11 (4-33), N = 17 and control: 5.5 (1-9), N = 12, p = 0.001 (Kruskall-Wallis test); (Figure 2). Table 1 shows *p* values calculated from multiple comparisons of MN-Heps data on NP, as well as the progression of fibrosis (F0 to F4). Only the F4 group showed statistically significant differences (p < 0.05).

## Discussion

To the best of our knowledge, this is the first study on chromatin loss, evaluated by MN formation, in liver samples from patients with CH/HCV during the fibrosis progression. A retrospective study of serial sections enabled us to evaluate CHP samples, which, through being particularly homogeneous, assured the detection of MN-Hep frequency. The results are consistent with the hypothesis that cytogenetic instability is already present in the initial stages of fibrosis, although the alterations in CHP (F1, F2, F3) differed neither mutually nor significantly, not even in those without fibrosis (F0) or in NP. On the contrary, the rate of MN-Heps in cirrhotic parenchyma (F4) was higher and different (p < 0.05) from F0, F1, F2 and F3, even though 93%-100% of the F1, F2, F3, F4 analyzed cases were with A1 and A2 inflammatory activity. Due to cirrhosis complexity, it is suggested that the long period of inflammation, inherent in HCV, may have caused an increase in proliferation, this contributing to the formation of MNi. Most of the MNi found in all cases of CH/HCV were medium to large sized, as already described in our previous work on HCV cirrhosis (Almeida *et al.* 2004). Oxidative and nitrosactive stress in CH/HCV (Choi and Ou, 2006; Seronello *et al.*, 2007) may cause both genotoxicity and, mutagenicity in hepatocytes. This phenomenon may create chromosomal instability (MN formation) in the diverse stages of fibrotic development in various ways (clastogenicity - chromatid breaks and/or aneugenicity - chromosome loss). The formation of MN by clastogenicity mechanisms has already been shown in genotoxic-induced hepatomas (Canonero *et al.*, 1997; Sanyal *et al.*, 1997), as well as in the evaluation of aneugenic substances (Fimognari *et al.*, 1997). The loss of spindle assembly checkpoints often occurs with a high frequency in HCC with chromosomal instability (Saeki *et al.*, 2002). The formation of MNi originating from several chromosomes was also obtained in human hepatoma HepG2 cells transfected with hepatitis B virus (HBV)-X genes, thereby providing evidence of alterations in genomic integrity (Livezey *et al.*, 2002). Studies of liver-cell lines showed that overexpression in hepatitis C virus NS5A protein is also directly involved in the formation of chromosome instability by mitotic cycle disregulation (Baek *et al.*, 2006). Thus, it is possible that the hypercarcinogenic state, due to chronic HCV infection (Hino *et al.*, 2002), might be associated with several genetic and epigenetic events, thereby explaining the presence of different-sized MN. Nevertheless, there was no relationship between MNi detected during fibrosis progression and changes in CHP morphological characteristics. We consider that the offset and increase in premature cytogenetic instability may play an important role in the innumerable lesions arising during CH/HCV evolution towards F4 (cirrhosis). A further conclusion of this work concerns the apparent cytogenetic normality of fibrotic parenchyma (F1-F3) in spite of chromosome loss evidenced by MN formation in liver hepatocytes during the development of CH/HCV.

## Figures and Tables

**Table 1:** *P* values calculated from multiple comparisons of MN-Heps data on the progression of fibrosis levels F0 to F4 and normal parenchyma (NP).

Groups	NP	F0	F1	F2	F3
F0	0.9998	-	-	-	-
F1	1.0000	1.0000	-	-	-
F2	0.9795	1.0000	1.0000	-	-
F3	1.0000	0.9934	0.9965	0.9531	-
F4	< 0.0001	0.0025	0.0232	0.0009	< 0.0001

Significance set at p < 0.05.
